# German and English Medical Education

**Published:** 1913-10-18

**Authors:** 


### The Hospital

The Workers' Newspaper of Administrative Medicine and Institutional Life, Administration, National Insurance and Health.

No. 1425, Vol. LV. SATURDAY, OCTOBER 18, 1913. PRICE ONE PENNY.

### GERMAN AND ENGLISH MEDICAL EDUCATION.

On more than one occasion during the past few years, when discussing rival plans of medical education, both British and foreign, we have emphasised very strongly the overwhelming advantages of the actual study of cases in the wards over all other aids to proficiency in the art and science of medicine. Our touchstone of a good medical school has been, we may say, the extent to which such facilities are offered to and accepted by the student; a criterion which we have been led to adopt by noting the types of practitioner which emerge from the various educational centres. Text-books, lectures, demonstrations, laboratories, and the rest of the complicated apparatus of medical education, all have their places; some, in our belief, are of more value than others, but all are essential to a complete and perfect school. Nevertheless, one and all of these are inferior, and should properly be regarded as secondary, to opportunities of actual bedside observation; and these should be so extensive as to permit each student being allotted such a number of cases, to be his exclusively for the time being, as to occupy the whole of his energies during his tenure of the usual clerkships and dresserships.

Formal lectures are, in our experience, of infinitely less value than is attached to them in some of our British schools, and *a fortiori* than on the Continent. One reason for this may be, probably is, the vastly greater output and superior quality of text-books nowadays, compared with a score or more of years ago. Semi-formal or totally informal clinical demonstrations in the wards are more likely to succeed; their value varies according to the capacity and character of the demonstrator, and this variation is an exceedingly wide one. As for compulsory attendance at a specified proportion of lecture-theatre orations as a necessary preliminary to qualification, the system seems to us thoroughly vicious and stupid, though it still exists in practically all our schools to some extent.

Holding such views, already several times expressed at greater length in these columns, we welcome very heartily the important letter from Dr. H. L. Tidy which was published in *The Times* on October 1, the opening day of the medical schools' year. The letter expresses our point of view with a virility and a directness which make it most attractive reading; and we sincerely trust that it will have weight with those who are being importuned to recast our system of medical training on Continental lines. Dr. Tidy is no blind optimist, convinced that British schools are perfect and foreign ones inferior; he recognises our defects and shortcomings in many respects, but he does plead for the retention of that peculiarly practical training which our students do get—and, let us add, more abundantly in London than even elsewhere in these islands. "Everyone will agree," says Dr. Tidy, "that the teaching of scientific subjects, and especially opportunities for research, are deficient in England. . . . It is here that great improvements may be made. The British methods of clinical teaching need protection. . . . Here and there a model clinic may be found abroad. Usually the modelness is rather from the point of view of the professor than of the student. It will not be easy to uphold these British methods against the schematic arrangements of Germany and Austria, with their powerful support in England. The tendency is towards set lectures at fixed hours on fixed subjects. Such an arrangement is beloved by a Government office. It is easily tabulated, printed, priced, and punched into statistics. A few teachers can handle a large number of students. The English clinician relies on the soundness of his bedside teaching. It is personal between the student, the patient, and the physician, and is the finest teaching in the world. The student is compelled to give his attention, otherwise he runs the risk of ridicule." With every word of which common-sense discourse we cordially agree.

Even more to the point, in view of the Teutonisation of our educational system which some of our critics advocate, is Dr. Tidy's plain speech on his first-hand observations of the German clinics and schools. Commissions and investigators, he complains, are often misled by the outward semblance of perfected organisation and the imposing time-tables of lectures into erroneous estimates of the actual work done. He quotes, as an example, the two world-famous clinics in Berlin. These are frequently spoken of as model clinics on an equality with each other; yet those who are intimately acquainted with them know that, though both are theoretically and superficially admirable, one is as good as a school can be, whereas the other is unsatisfactory. Whether the German system, an integral part of which, by the way, is the students' habit of wandering from one clinic to another, spending a term or two at each, is best adapted to the majority of the students of Germany is a large question, and needs no discussion in Britain. Certainly it is not the system which would answer best here; and we believe that it would prove a disastrous step to adopt it. By all manner of means let us keep our schools abreast of the times, let us improve the scientific side of our training, but do not let us abandon that purely clinical training at which we are pre-eminent; for it provides us with general practitioners second to none in the world. There is much else in Dr. Tidy's letter which must give every medical educationalist food for thought; but our immediate purpose has been to call attention to views which we have long held and are now exceedingly gratified to see so ably upheld and expounded by another. If we succeed in persuading any who have not read Dr. Tidy's letter to give it their serious consideration, we shall not be ill-content.

